# Long noncoding RNAs as promising biomarkers in cancer

**DOI:** 10.1016/j.ncrna.2022.02.004

**Published:** 2022-02-25

**Authors:** Ozal Beylerli, Ilgiz Gareev, Albert Sufianov, Tatiana Ilyasova, Yang Guang

**Affiliations:** a Federal Center of Neurosurgery, Tyumen, Russia; b Department of Neurosurgery, Sechenov First Moscow State Medical University (Sechenov University), Moscow, Russia; c Department of Neurosurgery, The First Affiliated Hospital of Harbin Medical University, Harbin, China; d Institute of Brain Science, Harbin Medical University, Harbin, China; e Bashkir State Medical University, Ufa, Republic of Bashkortostan, Russia

**Keywords:** Cancer, Exosomes, lncRNAs, Pathogenesis, Biomarker

## Abstract

Despite many advances in diagnosis and therapy (surgery, radiation therapy, chemotherapy), cancer remains one of the most important public health problems worldwide. Every day, the role of exosomes in cancer development and metastasis is being better described. Liquid biopsy was developed for early detection of cancer through minimally invasive and serial examinations of body fluids, with the advantage of tracking tumor progression in real time. Exosomes are extracellular membrane vesicles with a diameter of 30–100 nm, which are secreted by various types of cells and are present in most biological fluids. For a long time, they were considered non-functional cellular components, and today it has already been proven that they are a means of intercellular information exchange. They can move bioactive molecules such as proteins, lipids, RNA and DNA. Several studies have shown that their contents, including proteins and noncoding nucleic acids, may be of particular interest as biomarkers of diseases. The vast majority of gene transcripts are actually characterized as noncoding RNAs (ncRNAs) and are clusters of RNAs that do not encode functional proteins. They can be small, about 20 nucleotides in length, and are known as microRNAs (miRNAs), or transcripts over 200 nucleotides in length, defined as long noncoding RNAs (lncRNAs).

LncRNAs are a large group of ncRNAs over 200 nucleotides in length. LncRNAs, as regulatory factors, play an important role in complex cellular processes such as apoptosis, growth, differentiation, proliferation, etc. Recently, the results of many studies have also shown their essential role in carcinogenesis. Endogenous lncRNAs can be secreted by tumor cells into human biological fluids in the form of microvesicles, exosomes, or protein complexes, thereby forming circulating lncRNAs that are not degraded by RNA and are in a stable state. Aberrant expression of lncRNAs has been observed in cancer patients. In this context, endogenous lncRNAs can regulate the basic characteristics of cancer cells by controlling the expression of oncogenes associated with their suppressive and oncogenic functions. Therefore, circulating lncRNAs can be excellent biomarkers in cancer as well.

This paper provides an overview of current research on the functional role of lncRNAs in cancer and their potential clinical applications as diagnostic biomarkers and therapeutic targets for cancer.

## 1 Introduction

According to the 2016 American Cancer Statistics Reports, cancer has become a major public health problem in the United States and worldwide and is likely to surpass heart disease as the leading cause of death in the coming years [1]. The causes of death from cancer are numerous, while early diagnosis is the key to early detection and therapy. Currently, for early diagnosis, such research methods as X-ray, ultrasound, computed tomography (CT), magnetic resonance imaging (MRI) and endoscopy are used. However, there are many limitations. Since 1978, when Gerberman introduced the concept of a tumor marker, more than 100 tumor markers have been discovered that offer new ways of early diagnosis [2]. With the continuous development of molecular biology and histology, the study of tumor markers has become an important area in cancer diagnosis. In recent years, it has been established that long noncoding RNAs (Long noncoding RNAs, lncRNAs) play an important role in many biological processes, including gene regulation [3], cell cycle checkpoints [4], and cell migration [5]. Their role in the occurrence and development of cancer has already been proven. At the same time, the use of circulating LncRNAs in the diagnosis and treatment of cancer is attracting more and more attention from researchers and clinicians (see Table 1).

**Table 1:** The main technology of circulating lncRNA and its advantages/disadvantages.

Method	Advantage	Disadvantage
Microarray	High-throughput screening	High-cost, large-scale data, complicated operation, low sensitivity and specificity
Quantitative RT-PCR	Simple operation, low-cost	Low-throughput, low pecificity
Northern blotting	Low-cost, wide application	Complicated operation, low sensitivity, harmful experimental supplies
RNA sequencing	Time-saving, comprehensive, precision	High-cost, large-scale sequence data, not applicable for single gene
*In situ* hybridization	Spatial-and tissue-specificity	Low sensitivity, low specificity

Circulating exosomal lncRNAs are attracting the attention of cancer researchers. Exosomal lncRNAs can travel to recipient cells, where they transmit phenotypic changes. It was found that lncRNAs derived from exosomes are involved in tumor growth, metastasis, angiogenesis, and chemoresistance. In addition, exosomal lncRNAs can reprogram cells in the tumor microenvironment, thereby facilitating tumor development. Once inside exosomes, lncRNAs can be secreted into various body fluids [6]. LncRNAs in exosomes are protected from ribonuclease-mediated degradation and are stably present in body fluids [7]. Exosomal lncRNAs may have potential as biomarkers for various types of cancer [8].

In this review, we summarize current knowledge about the contribution of circulating exosomal lncRNAs to cancer progression and discuss their potential applications as novel biomarkers and therapeutic targets in cancer treatment.

### 1.1 LncRNA biogenesis

**Fig. 1:**
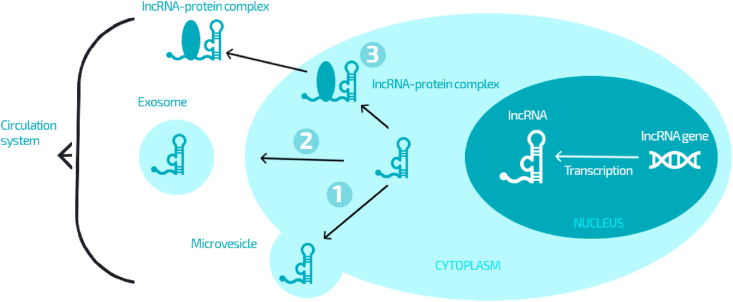
The origin of the circulating lncRNA. LncRNAs are secreted into the circulation system via three potential pathways. ①: microvesicle; ②: exosome; ③: binding with RNA-binding protein.

LncRNA is a kind of RNA molecule with a transcription length of more than 200 nucleotides and its own non-coding protein, which was originally considered a by-product of RNA polymerase II transcription, a “noise” of genome transcription and had no biological function [9,10]. However, studies in recent years have shown that lncRNAs can influence the level of gene expression at various levels through various mechanisms, and more and more studies show that lncRNAs play an important role in the onset, development and metastasis of tumors [11,12]. In the 1980s and 1990s, research reported the existence of circulating RNA, which laid the foundation for later lncRNA research. Due to RNA instability and easy degradation of ribonuclease in the blood, the mechanism by which lncRNAs are released into the blood and stabilized is not yet fully understood. Combined with existing research and analysis, sources of circulating lncRNAs could come from living cells (Fig. 1): 1), i.e. circulating lncRNAs can come from active secretion processes of living cells, most often in the form of exosomes and microvesicles. Despite the presence of ribonuclease in the blood, lncRNAs are still stabilized, mainly due to the protection of exosomes or microvesicles. Dong et al. tested the RNA content of blood microvesicles and found that lncRNAs in the blood are mainly distributed in exosomes, which also indicates that lncRNAs can be released into the blood through this form of extracellular vesicles [13]. At the same time, circulating lncRNAs can also form complexes associated with Ago2 proteins such as microRNAs (miRNAs), which are stabilized in the circulation like other non-coding RNAs. This means lncRNAs can enter the circulatory system by binding them to other secretions. 2) From apoptotic or necrotic cells [14]. There is a class of metastatic tumor cells in the primary or metastatic oven, which makes it more susceptible to invade the blood vessel endothelium and enter the circulation, called the circulating tumor cell (CTC) [14]. The survival time of CTC, as a rule, does not exceed 24 h after it has been injected into the blood. Tumor cells entering the blood, as a rule, retain specific markers of tissues of origin, i.e. epidermal markers. Analysis of peripheral cells in cancer patients and controls showed that mRNA in the blood of cancer patients ranged from 36% to 100%, suggesting that LncRNAs may come from circulating tumor cells invading blood vessels [15,16].

### 1.2 Circulating lncRNAs as cancer biomarkers

Tumor antigens and ectopic hormones are currently commonly used clinically used tumor markers. For example, cancer embryonic antigen (CEA) is used for early warning of colorectal and breast cancer, and alpha-fetoprotein (AFP) is used for early warning of stomach, colon, etc., and these traditional biomarkers are widely used in early diagnosis tumors [17,18]. However, traditional markers have many disadvantages, including specificity and sensitivity is not high, the same mark can predict different cancer risks, but also have a high chance of missed and misdiagnosis [[19], [20], [21]]. LncRNAs have been found in body fluids such as plasma, serum, urine, etc. using real-time PCR. Thus, circulating lncRNAs are becoming the focus of new tumor markers [22]. Previously detected lncRNA H19 is closely associated with various tumors, and in 32 blood samples from gastric cancer patients and healthy people, it was found that H19 is significantly higher expressed in gastric cancer patients, and there is a more obvious association with pathological factors such as patient survival [23]. As a biomarker, circulating lncRNAs can stably exist in the circulatory system and have properties free from nuclease degradation [24]. At the same time, circulating lncRNAs can be found in body fluids such as blood, urine, etc. Thus, circulating lncRNAs have a simple and rich source, making them promising molecules of high diagnostic value.

### 1.3 Circulating lncRNAs in cancer diagnostics

Since circulating lncRNAs can be accurately detected, they can be used as a novel biomarker for tumor diagnosis. Currently, they are used in the clinical diagnosis of various tumors by detecting the expression level of lncRNAs.

#### 1.3.1 Lung cancer

The incidence and mortality rate of lung cancer ranks first among malignant neoplasms. A significant proportion of patients were diagnosed at late stages with a poor prognosis and a five-year survival rate of less than 15%. Currently, the technology for early detection of lung cancer is constantly evolving, and the search for new cancer markers is urgent. Circulating lncRNA as a minimally invasive biomarker has been proven in lung cancer. Weber et al. examined blood samples from 45 patients with Non-small cell lung cancer (NSCLC) and 25 healthy volunteers to detect lncRNA MALAT 1 expression to determine its value as a marker [25]. The results showed that it was significantly highly expressed in patients with NSCLC, the area under the ROC curve (Area under ROC curve, AUC) at the level of 0.79, indicating that circulating MALAT1 lncRNA is of high value as a diagnosis of NSCLC, and its minimally invasiveness and specificity indicate that MALAT1 can be used as a novel tumor marker. Hu et al. screened for new markers by detecting lncRNAs that are abnormally expressed in the plasma of NSCLC patients [26]. They found that the 3 lncRNA SPRY4-IT1, ANRIL and NEAT1, have high sensitivity and specificity in NSCLC. Analysis of their diagnostic value yielded AMC values of 0.603, 0.798, and 0.693, respectively, while co-testing of the three lncRNAs showed higher sensitivity and specificity, with an AMC of 0.876. These results indicate that these three lncRNAs are expected to be novel markers for diagnosing NSCLC. Liang et al. found that GAS5 lncRNA in NSCLC tissues showed differential expression through detection in plasma samples from 90 NSCLC patients and 30 healthy volunteers [27]. lncRNA GAS5 and NSCLC stage have a very strong relationship (P|0.024), in patients with grades III and IV, expression is significantly lower than I and II. GAS5 has an AMC value of 0.832 (P < 0.000 1; sensitivity 82.2%; specificity 72%), suggesting that it can be used to differentiate patients with NSCLC from the general population, in conjunction with its use with the commonly used currently CEA, and the AUC value reaches 0.909. All of this speaks to the potential value of GAS5 as an NSCLC biomarker. To sum up, there is a difference in the circulatory system, which expresses that lncRNA is associated with various pathological factors in patients with lung cancer and is expected to serve as a new biomarker for diagnosing lung cancer.

#### 1.3.2 Breast cancer

Breast cancer is a malignant tumor that originates in the epidermal tissue of the breast, the incidence and mortality of which has increased dramatically in recent years [28]. The use of circulating lncRNAs in the diagnosis of breast cancer has already been studied. Xu et al. studied sera from 68 breast cancer patients and 68 healthy volunteers using RT-PCR and found that lncRNA RP11-445H22.4 expressions was significantly higher in the serum of breast cancer patients [29]. In order to better understand the clinical value of lncRNA, the investigators further compared the ROC and AMC values of lncRNA with common tumor serum markers such as AFP, CEA, CA125 and CA153 and showed that lncRNA RP11-445H22.4 has the highest sensitivity and specificity. In addition, lncRNA has a higher sensitivity compared to traditional ultrasound diagnostic methods. These results indicate that lncRNA RP11-445H22.4 could be a new potential biomarker for breast cancer. Miao et al. examined 78 tissue samples from breast cancer patients to detect the expression of lncRNA MALAT1 and found that its expression is significantly higher in cancer tissues, and lncRNA MALAT1 in breast cancer tissues is associated with lymph node metastases [30]. At the same time, in serum samples from patients with breast cancer, lncRNA MALAT1 is expressed significantly higher than in benign breast diseases. Zhang et al. used the same method to determine differentiated H19 lncRNA expression in cancer tissues and then tested their expression in serum samples from both 96 healthy (control) and 102 breast cancer patients and found their significantly higher expression (P < 0.05); its ACC is 0.81, which is higher than CEA and CA153 [31]. In addition, experiments showed that lncRNA H19 expression in the postoperative serum sample was significantly lower than preoperative levels (P|0.000 6), suggesting that H19 could be used for pre-testing in addition to being a breast cancer marker.

#### 1.3.3 Gastric cancer

Gastric cancer incidence and mortality is the second leading malignancy, and delays in diagnosis and limited treatments make gastric cancer an important clinical problem. Hashad et al. showed that lncRNA H19 has a specific expression in gastric cancer [23]. After testing 32 blood samples from healthy volunteers with 32 cases of gastric cancer, lncRNA H19 expression levels were significantly higher in patients with gastric cancer and had nothing to do with gender and age. In combination with CAI analysis of the tumor serum marker currently in use, it was found that lncRNA H19 expression level was associated with CAI level, while ROC curve analysis showed that H19 lncRNA was better than CAI as a biomarker. The combined analysis of CAI and H19 lncRNA is used to diagnose gastric cancer and their ACC value was further increased to 80.4%. These results indicate that H19 lncRNA is a very valuable potential new marker for gastric cancer and co-diagnosis with CAI will yield better results. Jin et al. through 173 gastric cancer patients (including 100 early stage gastric cancer patients, 62 operated patients, and 11 relapses with blood sample analysis), 30 intestinal polyps, 30 cases of atypical hyperplasia consistent with blood samples from healthy volunteers, to test lncRNA HULC expression [32]. It was found that compared with healthy individuals and patients with common gastric diseases such as intestinal polyps, HULC of gastric cancer patients showed significantly higher expression. In addition, comparison of HULC (AUC|0.888) and CEA (AUC|0.694), CA72-4 (AUC|0.514) and other commonly used serum markers showed that HULC has more effective diagnostic capabilities. Dong et al. analyzed 65 cancer-associated lncRNAs [33]. They were filtered in the LncRNA-Disease association database with the keyword “cancer” and 39 lncRNAs were further verified through the Refseq database on the NCBI website. LncRNAs, which had statistically significant differences in 40 pairs of cancer tissues, were then tested in blood samples from 10 patients and 10 healthy volunteers. Ultimately, lncRNA CUDR, LSINCT-5, and PTENP1 showed better diagnostic performance when used together (AUC|0.92). Compared to the commonly used blood gastric cancer markers CAA and CA19-9, the diagnostic value is higher, indicating that these three serum lncRNAs are expected to be better diagnostic markers of gastric cancer than CAA and CA19-9. In summary, in the diagnosis of gastric cancer, circulating lncRNA has shown its application value in numerous studies and can play a large role in combined diagnostics, providing new approaches for future innovative diagnostic methods of gastric cancer.

#### 1.3.4 Liver cancer

Hepatocellular carcinoma (HCC) is the second most common worldwide. Wang et al., by analyzing the expression of lncRNAs in the blood, found that the expression of lncRNAs in patients with liver cancer is significantly higher than in patients with hepatitis B and the healthy group [34]. The lncRNAs uc003wbd and AF085935 can be used to differentiate liver cancer from hepatitis B and healthy individuals, suggesting that these two lncRNAs are of great diagnostic value. At the same time, Lu et al. found that lncRNA uc003wbd and lncRNA-AF085935, in 137 cases of HCC patients, 104 patients with hepatitis B and 138 healthy volunteers for a total of 379 serum samples, were significantly higher than in healthy volunteers and patients with hepatitis B [35]. Further analysis of the ROC curve shows that, in contrast to patients with HCC and hepatitis B, lncRNA uc003wbd has an ACC value of 0.70 and lncRNA AF085935 has an AUC value of 0.86. With a difference between HCC patients and health controls, the AUC values of the two lncRNAs were 0.86 and 0.96, respectively. Thus, both lncRNAs could become new serum HCC biomarkers. Yu et al. believe that the distinctiveness and sensitivity of markers is not particularly good if they are built on only one tumor-associated lncRNA [36]. If different lncRNAs-associated tumors in combination can significantly improve the diagnostic value of the marker, they found that PBT1 and uc002mbe can be used to differentiate between HCC patients and the healthy group, the AUC value is 0.764 (95% CI: 0.648–0.833), which is better, than a traditional AFP marker and therefore has a very high application value. The above results indicate that various circulating lncRNAs have the potential to become novel liver cell cancer markers and will be widely used.

#### 1.3.5 Prostate cancer

Prostate cancer is a very common type of cancer in men and is on the rise worldwide. Currently, early diagnosis of prostate cancer mainly depends on the detection of prostate-specific antigens (PSA) in the blood. LncRNA PCA3 is an lncRNA that is currently well studied in prostate cancer, is significantly higher expressed in prostate cancer tissues, and can be detected in urine as an early molecular diagnostic marker [37]. Feibus et al. have shown that lncRNA PCA3 has a significantly higher urinary expression in patients with prostate cancer [38]. Isin et al. have shown that lincRNA-p21 can be used to differentiate prostate cancer from benign prostatic hyperplasia (BPH) [39]. They tested LincRNA-p21 expression in urine samples from 30 prostate cancer patients and 49 BPH patients, found that lincRNA-p21 was significantly higher in the urine of prostate cancer patients, and further compared the ROC curve used by LincRNA-p21 and its combined with the common serum marker PSA, and found that the specificity increased significantly after testing lincRNA-p21 and PSA together, but the sensitivity was not significantly changed. Summing up, it can be noted that circulating lncRNAs have a wide application value in the early diagnosis of common malignant neoplasms and can develop into a new type of biomarker.

## 2 Conclusions

Cancer is still a major problem for people and early diagnosis plays a very important role in treatment and many cases can be completely cured if patients are found early. Nowadays, with the continuous progress in the field of molecular biology and high throughput sequencing technology, more and more attention is being paid to lncRNAs, which were previously called gene “junk”. At the same time, numerous studies show that lncRNAs play an important role in the regulation of gene expression and between growth, proliferation, apoptosis and cellular communication of cells, and studies now show that lncRNAs have different levels of expression change in tumors of any localization [40,41]. Biological processes such as invasive migration are closely linked. Circulating LncRNAs have been found to be stable in blood or other body fluids (urine, saliva, cerebrospinal fluid, etc.), which makes it easy to detect them [42]. Circulating LncRNAs, as a kind of molecular marker with lower specificity and sensitivity than traditional tumor markers, are attracting more and more attention.

## Funding

This work was supported by 10.13039/501100001809National Natural Science Foundations of China (81971135); Natural Science Foundations of Heilongjiang (YQ2020H014); “Chunhui Plan”of Ministry of Education (HLJ2019009); Distinguished Young Foundations of the First Affiliated Hospital of 10.13039/100010722Harbin Medical University (HYD2020JQ0014);

## Ethical approval

This article does not contain any studies with human participants performed by any of the authors.

## Declaration of competing interest

The authors declare they have no conflict of interest.
